# The importance of the gut microbiome in the pathogenesis and transmission of SARS-CoV-2: Someone on Earth: “..*we moved at the speed of Science*!” - Science from the center of the Universe: “*Hey man, I’m still waiting for you in the 50s!*”

**DOI:** 10.1080/19490976.2023.2244718

**Published:** 2023-08-09

**Authors:** Carlo Brogna, Valentina Viduto, Mark Fabrowski, Simone Cristoni, Giuliano Marino, Luigi Montano, Marina Piscopo

**Affiliations:** a Department of Research, Craniomed Group Facility Srl, Bresso, Italy; b Long COVID-19 Foundation, Brookfield Court, Leeds, Garforth, UK; c Emergency Department, University Hospitals Sussex, Brighton, UK; d Department of Chemistry, ISB – Ion Source & Biotechnologies Srl, Bresso, Italy; e Marsan Consulting Srl., Public Health Company; via Dei Fiorentini, Napoli, Italy; f Andrology Unit and Service of LifeStyle Medicine in Uro-Andrology, Local Health Authority (ASL) Salerno, Salerno, Italy; g Department of Biology, University of Naples Federico II, Naples, Italy

**Keywords:** MIS-C, COVID-19, gut microbiome, SARS-CoV-2

## Abstract

Zhou et al. study nicely traces a significant topic in COVID-19 infection: the persistence of the virus within the intestinal tract. Many pathological mechanisms have been noted in the current literature about the mode of infection and propagation of SARS-CoV-2 in the human body. Nevertheless, there are still many concerns about this: only some things seem well understood. We present a different point of view by illustrating the importance of the gut microbiome in the pathogenesis of COVID-19 disorders.

Dear Editor,

We read with interest the perspective by the authors in^1^ in the journal Gut.

Their suggestion for the persistence of the virus (SARS-CoV-2) in the gastrointestinal tract is very important, and this is the same finding observed by other authors too.^2^ Their paper aligns with what has already been reviewed by Davis et al.^2^ With this regard, it should be pointed that the authors in^1–3^ highlight the persistence in the gastrointestinal tract of the SARS-CoV-2 virus and the associated dysbiosis. It is critical to emphasize a key element.

The following results, *the bacteriophage behavior*, reported in the papers^4,5^ show how RNA of SARS-CoV-2 can replicate in bacteria through mass spectrometry, genetic testing, microscopy, and the nitrogen isotope ^15^N (Figure 1c).

**Figure 1.**
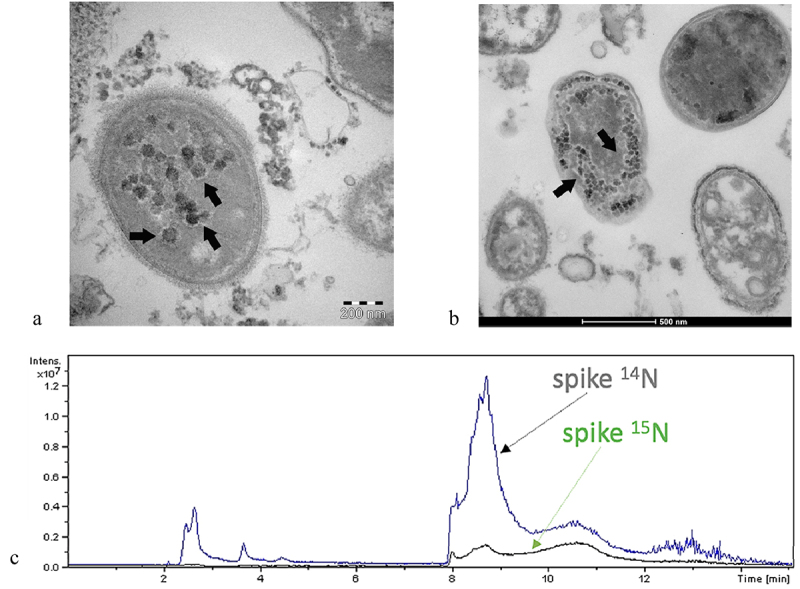
The bacteriophagic behavior of SARS-CoV-2. Panels a and b: Transmission electron microscope images (panels a and B, TEM FEI, Thermo Fisher Tecnai G2 operating at 120 kV) show SARS-CoV-2 (indicated by black arrows) inside two bacteria. Panel C: The proteomic profile at mass spectrometry confirms the presence of an abundance of SARS-CoV-2 proteins; Peptide mapping of SARS-CoV-2 spike protein was acquired by means of liquid chromatography-mass spectrometry associated with ^14^N and ^15^N profiles and performed on an aliquot of bacteria, derived from the human gut microbiome, culture after 7 days with the presence of SARS-CoV-2^4–10^. Images obtained for the gentle concession of the authors^4–10^.

Interesting is the involvement of *toxin-like peptides* by gut microbiome during acute stress caused by viral persistence and how this phenomenon persists over time.^4–10^ In the in vivo model, we have to take into account the interactions and exchanges of peptides that occur between the overall ecosystem and entire microenvironment, and how the immune system can be modulated by them; the presence of these peptides, called toxin-like emphasizes how complex the microenvironment under consideration is and hints at possible mechanisms of exchange between different animal kingdoms.^11–16^

What the authors state in^1–3^ are substantial and need a comprehensive overview of the mechanisms so that the scientific and medical community can fully assess the virulence actions of SARS-CoV-2. This impacts the progress and direction of research and potential treatment modalities, including recommendations on other ways to develop vaccines.

The importance of viral persistence in the gut microbiome could be critical to consider. Viral persistence, gut dysbiosis, never-before-observed toxin production, and other possible treatment modalities we have suggested are significant evidence that deserves to be included in the scientific arguments. The studies cited highlight clear evidence of RNA virus replication within bacteria, which seems no one has clearly demonstrated in 80 years of medical history. Fauci et al.^17^ have an afterthought on the replication dynamics of respiratory viruses with mucous membranes as their preferred area. It is not difficult to remember that the realm of bacteria lies above this anatomical district. These new observations invite a revision of current microbiology and virology knowledge and prompt us to review other historically known viruses.

It should be emphasized that knowledge of a different replication and propagation mechanism forces us to rethink the most suitable type of vaccination, considering that the microbiome can also take on a resistance function against the viral pathogen. In light of these data, the importance of attenuated oral vaccination of a favorable strain, like the one that was successful in Dr. Sabin’s and polio time, emerges. Considering the involvement of the gut microbiome could be possible also to explain the success of Sabin’s vaccinology over injection methods against poliovirus (PLV). Repeating the same experiments summarized here also for poliovirus could bring to light a mechanism that has always been present but never analyzed before.

A suggestion arises that should be applied during a pandemic, based on the consideration that our planet is based on an ecosystem built over millions of years with the dominant species being bacteria: “It is appropriate for scientists, *regardless of how viral pathogen originated, to consider interactions with prokaryotic cells before* defining an exceptional virus that infects the mammalian cell, independently.”
